# Ultrafast Laser Manipulation of In‐Lattice Plasmonic Nanoparticles

**DOI:** 10.1002/advs.202402840

**Published:** 2024-07-18

**Authors:** Han Zhu, Lingrui Chu, Hengyue Lv, Qingchuan Ye, Saulius Juodkazis, Feng Chen

**Affiliations:** ^1^ School of Physics State Key Laboratory of Crystal Materials Shandong University Jinan 250100 China; ^2^ Optical Sciences Centre Faculty of Science Engineering and Technology Swinburne University of Technology Hawthorn VIC 3122 Australia

**Keywords:** hybrid plasmonic materials, optical forces, photoluminescence, surface plasmons, third‐order nonlinearity

## Abstract

Plasmonic nanoparticles enable manipulation and enhancement of light fields at deep subwavelength scales, leading to structures and devices for diverse applications in optics. Despite hybrid plasmonic materials display remarkable optical properties due to interactions between components in nanoproximity, scalable production of plasmonic nanostructures within a single‐crystalline matrix to achieve an ideal plasmon–crystal interface remains challenging. Here, a novel approach is presented to realize efficient manipulation of in‐lattice plasmonic nanoparticles. Employing ultrafast‐laser‐driven plasmonic nanolithography, metallic nanoparticles with controllable morphology are precisely defined in the crystalline lattice of yttrium aluminum garnet (YAG) crystal. Through direct ion implantation, hybrid plasmonic material composed of nanoparticles embedded in a sub‐surface amorphous YAG layer is created. Subsequently, femtosecond laser pulses guide formation and reshaping of plasmonic nanoparticles from the amorphous layer into the single‐crystalline matrix along direction of light propagation, facilitated by a plasmon‐mediated evolution of laser energy deposition. By tailoring resonance modes and optimizing the coupling between structured particle assemblies, a range of applications including polarization‐dependent absorption and nonlinearity, controllable photoluminescence, and structural color generation is demonstrated. This research introduces a new approach for fabricating advanced optical materials featuring in‐lattice plasmonic nanostructures, paving the way for the development of diverse functional photonic devices.

## Graphical abstract

A sophisticated plasmonic nanolithography process for functional crystals has resulted in successful fabrication of nanorods exhibiting robust polarization selectivity directly within the lattice. Several applications are demonstrated through on‐demand writing of plasmonic nanoparticle arrays. This approach harbors the capability to tailor on‐chip/in‐chip integrated micro‐nanophotonic structures and devices with exceptional properties in functional materials/single crystal films.

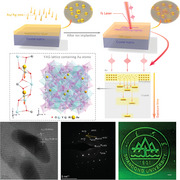

## 1 Introduction

Surface plasmon nanomaterials, exemplified by noble metal nanoparticles (NPs), have gained widespread applications in nanophotonics due to their exceptional capability to interact with electromagnetic fields beyond the diffraction limit.^[^ ^1^, ^2^, ^3^, ^4^, ^5^, ^6^ ^]^ The high optical cross‐section produced by the localized surface plasmon resonance (LSPR) effect allows NPs to increase the optical scattering rate of adjacent materials or to induce localized heating, which is the basis of technologies including surface‐enhanced Raman spectroscopy,^[^ ^7^, ^8^, ^9^ ^]^ biochemical sensing,^[^ ^10^, ^11^, ^12^ ^]^ and plasmonic photothermal conversion.^[^ ^13^, ^14^ ^]^ As nanophotonics advances, hybrid plasmonic nanostructures—typically consisting of aligned NPs with attached non‐plasmonic materials—have exhibited novel physical properties in optics and optoelectronics attributed to unique bonding modes and energy/carrier flow mechanisms.^[^ ^15^, ^16^, ^17^, ^18^, ^19^, ^20^, ^21^ ^]^ Through precise design of nanoparticle assemblies and utilization of atomically contacted non‐plasmonic components, near‐field light customization and functional devices of exceptional efficiency can be realized. Despite the evident advantages, practical applications of NP assembly for light field modulation remain limited. The challenge is not only to synthesize NPs of appropriate size and morphology, but more importantly, structural stability, scalable preparation, and customized dielectric environment. Moreover, long‐term chemical stability of nanomaterials is of paramount importance favoring inside‐volume (3D) rather on‐the‐surface (2D) solutions.

Functional surface plasmon structures for tailoring optical responses are generally manifest as metasurfaces directly defined on substrate surfaces.^[^ ^22^, ^23^, ^24^, ^25^, ^26^ ^]^ These orderly assembled nanoparticle arrays rely heavily on expensive and difficult‐to‐scale nanofabrication techniques, necessitating additional encapsulation processes to ensure stability. Furthermore, plasmon‐dielectric environment coupling is difficult to achieve for structures located on the surface. Recently, ultrafast laser processing, as a powerful tool for creating artificial microstructures in transparent materials,^[^ ^27^, ^28^, ^29^, ^30^, ^31^ ^]^ has shown promise in inducing and modifying NPs inside doped precursor glasses.^[^ ^32^, ^33^, ^34^, ^35^ ^]^ Additionally, nano‐layers can be utilized for laser‐induced recrystallization into spatially 3D localized NPs.^[^ ^36^ ^]^ Nanoparticle modification and the direct inscription of arrays can be carried out in micro/nanoscale regions through ion migration at the focal point or plasmon‐enhanced photothermal conversion. Nevertheless, the tunability of morphology and coupling to dielectric environments in functional structures remain somewhat limited. Particularly noteworthy is the absence of reported methods for fabricating plasmonic nanostructures within optical crystals, which hold the promise of offering diverse spectral and nonlinear functionalities.

In this work, we present a novel approach for the precise manipulation of plasmonic nanoparticle assemblies, allowing for their controlled formation directly within the crystal lattice using a plasmonic nanolithography technique involving a two‐step top‐down process. Initially, a modified layer is induced beneath the material's surface through ion implantation, followed by ultrafast laser irradiation that triggers highly localized light‐matter interactions, enabling patterning of NPs along the laser's propagation path. This method enables the synthesis of NPs with specific morphologies (size and shape) within the crystal structure. We showcase the successful fabrication of nanorods exhibiting robust polarization selectivity in a widely used, commercially available optical crystal of yttrium aluminum garnet (YAG), while maintaining the lattice's structural integrity. By fine‐tuning the laser processing parameters, we elucidate the evolution and mechanisms underlying nanoparticle formation within the crystal. Consequently, we achieve precise control over polarization, tunable plasmon resonances and nonlinearities, enhanced photoluminescence, and structural color through direct write nanolithography of plasmonic nanoparticle arrays.

We use YAG with embedded Au/Ag NPs in postimplantation substrates. The sub‐surface regions with NPs act as a precursor for subsequent laser manipulation (Table S1, Supporting Information). Despite the inherent lattice damage in the implanted zone leading to an amorphization and structural defect formation causing change of crystal's dielectric properties, this method remains the only feasible approach for precisely depositing NPs within solid materials along the axial direction at the nanoscale level. Subsequent exposure to ultrafast laser triggers plasmon‐mediated nanoparticle evolution, facilitating the tailored creation of nanostructures through parameter selection as well as reconstruction in the crystal host. This innovative strategy can be effectively applied to other optical crystals to establish localized surface plasmon resonance systems for tailor‐made optical responses.

## 2 Results and Discussion

### 2.1 Ex Situ Reconstruction of NPs in Crystals

**Figure 1:**
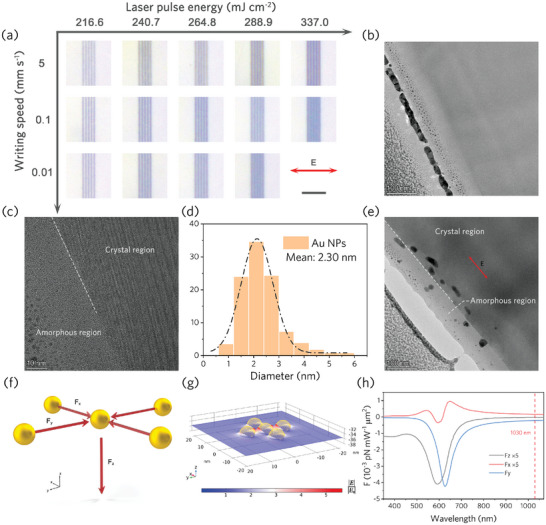
Femtosecond laser direct writing of YAG crystals containing AuNPs. a) Top optical microscope view of laser inscribed nanoparticle assembly arrays with different parameters buried in YAG. Scale bar: 10 µm. b) Cross‐sectional TEM image, c) HRTEM image of the YAG implanted layer, and d) corresponding Au NPs particle size analysis. e) Cross‐sectional TEM image of Au NPs in YAG after laser irradiation of 216.6 mJ cm^−2^ 5 mm s^−1^. f) Schematic of optical forces between NPs illuminated by light transmitted along the z‐axis. g,h) Numerical calculation of the near field and felt optical force of NPs in YAG. LSPR is excited by light (1030 nm) polarized along the y‐axis propagating along the z‐axis. *F*_x_/*F*_y_ are the forces along the x/y axis felt by particles adjacent to the central particle in the positive x‐axis direction and the negative y‐axis direction, respectively. *F*_z_ is the scattering force exerted on all NPs in the model.

The LSPR of NPs excited by ultrashort pulse irradiation will produce strong light absorption and scattering, which allows heating of electrons through Landau damping, direct energy deposition and redistribution through a short non‐radiative relaxation process, thereby driving the evolution of the irradiated region. We demonstrate this process using femtosecond laser pulses with a center wavelength of 1030 nm at repetition rate of 5 MHz, and control of the evolution and patterning of NPs system through the laser scanning speed and pulse energy, hence, via a controlled energy deposition. **Figure** **1**a shows arrays composed of NPs directly written by femtosecond laser with different parameters in YAG containing Au NPs. It can be seen that changes in pulse intensity and action time lead to color changes in the structures composed of NPs with different morphologies. The energy used for irradiation is much lower than the threshold required to write obvious traces of structural modification on the surface of pure YAG (Figure S1, Supporting Information), indicating that the modification results from plasmon‐enhanced energy deposition and redistribution. The morphology characterization of the unmodified area by transmission electron microscopy (TEM) and high‐resolution transmission electron microscopy (HRTEM) is shown in Figure 1b–d and Figure S2, Supporting Information. The ion implantation forms a uniform implantation layer containing high‐density NPs at the subsurface of YAG, which allows the energy captured from the incident field to be coupled to the entire system via intense near‐field and proximity plasmon hybridization, rather than being localized to a few nanometers around the particle.

It is revealed that the interaction of the laser pulse with the nanoparticle system results in the displacement of the NPs along the direction of light propagation and reshaping into nanorods along the polarization (Figure 1e). There is intricate interaction within an ensemble of NPs dependent on their separation and size‐shape via scattering and absorption cross sections. The resulting local electrical field (*E*) enhancement at a single particle depends on its own properties as well as nearest neighbors. The qualitative picture of such mechanism is captured by formation of bulk ripples: for an individual volume of metallic breakdown plasma (as a nanoparticle) it creates local enhancement in response to the scattered *E*‐field from neighboring nanostructures.^[^ ^37^, ^38^ ^]^ The difference is that nano‐plasma particles could have subcritical plasma density for the laser wavelength, while in the case of already present metallic NPs, the electron density in the nanoparticle is above the critical plasma density. This means a strong scattering including reflection from NPs. Strong reflection promotes linear deposition of laser beam momentum by definition, the photon pressure *P*_ph_ = *I*/c, where light intensity *I* ∼ *E* ^2^. Next more detailed estimates are made by simulations.

We introduced an optical force model to help understand this phenomenon (Figure 1f) and simulated the optical response of a scatterer composed of five NPs embedded in YAG crystal through the finite element method (Figure 1g,h). The optical forces acting on the structures are calculated by the Maxwell tensor method (see Experimental Section for more details). As an intuitive result of photon momentum transfer, electromagnetic waves propagating along the z‐axis will bring about a scattering force *F*_z_ that pushes the particles along the propagation direction (axially). Since the laser intensity at the focus has a Gaussian distribution, for our experimental pulse width of 350 fs and energy density of 216.6 mJ cm^−2^ (Figure 1a), the *F*_z_ felt by the irradiated NPs in the effective modification area (much smaller than the focal spot diameter 2.8 µm, see Experimental Section) during the pulse duration can exceed 34.6 pN, which is very high to drive nanoscale objects or atoms/clusters to move. In addition, interactions perpendicular to the propagation direction (laterally) can also occur between NPs (Figure 1f), the so‐called “plasmonic tweezers,”^[^ ^39^ ^]^ which originate from the potential well associated with the particle's near field (Figure 1g), causing NPs along the polarization direction (y‐axis) to attract each other while others mutually exclusive in one direction (Figure 1h and Figure S3, Supporting Information). Such lateral gradient forces are involved together with the nucleation‐growth‐Ostwald ripening of NPs in a positive feedback process that drives the in situ evolution of the system, as evidenced by the aggregation of NPs during plasmonic lithography of ion‐implanted glasses.^[^ ^34^, ^35^ ^]^

**Figure 2:**
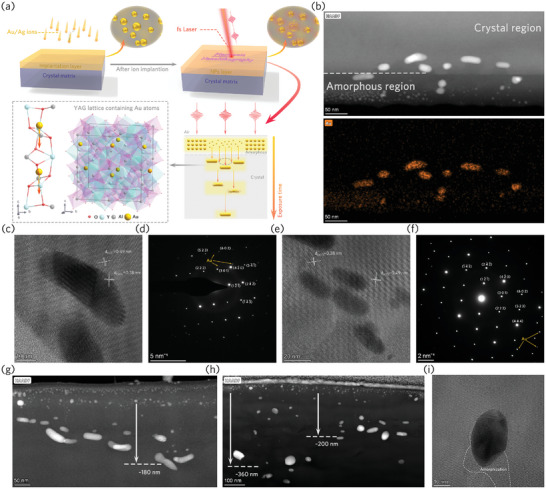
a) Schematic of the plasmonic nanolithography process of crystalline materials. b) HAADF and energy dispersive X‐ray spectroscopic mapping image of the cross‐section of the laser irradiated area, corresponding to the write parameter of 216.6 mJ cm^−2^ at a write speed of 5 mm s^−1^. c–f) Cross‐sectional HRTEM and SAED images of the single crystal region after laser irradiation with pulse energies of c,d) 216.6 and e,f) 264.8 mJ cm^−2^ and a scanning speed of 5 mm s^−1^. g,h) Cross‐sectional HAADF images of the modified area after laser irradiation with pulse energy of 264.8 mJ cm^−2^ and scanning speeds of 5 and 0.1 mm s^−1^, respectively. i) HRTEM image of Au NPs after laser irradiation with parameter 337.0 mJ cm^−2^ 0.1 mm s^−1^.

**Figure** **2**a illustrates the entire plasmonic nanolithography process for assembling NPs within the YAG lattice. The Au/Ag elements are incorporated into the target through an ion beam at a certain angle to the surface, and are distributed within the ion range due to the electron and nuclear stopping process, which results in an amorphous implanted layer (Figure 1c and Figure S3a–c, Supporting Information). This amorphized implant layer provides NPs and metal atom (and ion) concentration for subsequent processing steps. Subsequently, on‐demand writing of nanoparticle structures is achieved by direct femtosecond laser irradiation of the implanted layer. High‐angle annular dark field (HAADF) and energy‐dispersive X‐ray spectroscopic mapping shown in Figure 2b further confirmed that Au nanorods with long axes parallel to the polarization of the incident light were formed in the single crystal region after laser irradiation. The morphological structure of the modified area after processing with different laser parameters shown in Figure 2c–I and Figures S4–S10, Supporting Information, demonstrates this plasmon‐mediated evolution of the NPs system. As a direct result of laser energy deposition, the positive feedback mechanism generated by plasmon–matter interaction and light lateral force that first appear in the modified region will drive the agglomeration and growth of NPs in the implanted layer, mainly manifested as an increase in particle size (Figures S4b, S6b, S7c, and S9c, Supporting Information), which is consistent with the phenomenon observed in glass containing NPs. It is worth noting that, along with particle size changes (which means an enhancement of the LSPR effect), ultrashort pulse irradiation also results in intense ultrafast electron emission and couples energy to the constituent atoms within a few ps time.^[^ ^40^ ^]^ This process enables atoms or clusters to overcome surface tension and dissociate from the nanoparticle surface. Nanoparticle localized heat deposition and local melting is promoted as particles become larger which also case a stronger scattering (depends on volume), which contribute to momentum transfer (“photon wind”).

As nanoparticle grows and is lower than skin depth in Au, it acts as an absorbing dielectric nano‐sphere which refocus incoming light on the opposite side (qualitative phenomenon observed in perfect absorbers with light localization below thin nanoparticle.^[^ ^41^ ^]^ This could contribute to melting and axial push along the laser beam (which provides the photon pressure). High localized temperature around nanoparticle also contributes to recrystallization as metallic nano‐object moves through. This allows atoms or clusters carrying initial kinetic energy to diffuse in the direction of light propagation.^[^ ^42^, ^43^ ^]^

The amorphous layer (implanted layer) caused by ion implantation damage is very thin (≈100 nm), making it easy for migrating elements involved in ex situ reconstruction to pass through and enter the single crystal region. The atoms/ions/clusters that move a certain distance along the beam axis accumulate locally within the crystal until they surpass the solubility limit, subsequently precipitating and nucleating under the influence of thermal fluctuations. The metal‐crystal interface (which is also a heterojunction) is an active red‐ox site since polarization driven electrons are driven between the two materials.^[^ ^44^ ^]^ This promotes red‐ox reactions and implanted metal ions forms neutral atoms, which forms larger NPs at the tips of NPs where localized host melting/softening takes place under action of high‐intensity laser irradiation. In marked contrast to the amorphous lattice structure of glass, where only in situ evolution of the NPs occurs,^[^ ^34^, ^35^ ^]^ the intact crystal structure allows atoms to migrate through the channels and regenerate in areas where the crystal lattice is intact.

Benefiting from the highly localized near field, the photothermal conversion process initially only occurs in the amorphous region, and the single crystal part of the matrix is still “cool,” causing the nanoseeds of NPs to supersaturate and precipitate at the two poles along the polarization of the laser pulse to form nanorods. The red‐ox processes occur simultaneously at the interface along polarization driven electrons providing reduced metal atoms for NP growth and formation. The long axis length of the finally formed nanorods ranges from 14 to 80 nm at our experimental resolution. HRTEM and selected area electron diffraction (SAED) of the single crystal region after irradiation confirmed that the NPs are synthesized directly in the YAG lattice (Figure 2c–f and Figures S4, S6, and S7, Supporting Information), which provides the possibility for plasmon‐lattice coupling to achieve new physical properties. The lattice fringes in the regions shown in Figure 2c,e show interplanar spacings of 0.38 and 0.49 nm, which match well with the (301) and (121) planes of body‐centered cubic (bcc) YAG, respectively, and are consistent with the results shown in the SAED images. This interesting phenomenon of diffusional growth and push of metallic NPs long laser pulse propagation inside crystalline host calls for more detailed studies on surface energy effects and solid‐state epitaxy.^[^ ^45^ ^]^

It is obvious that increasing the pulse energy results in a further displacement of the NPs from the implanted layer toward the interior of the crystal, as this implies stronger photon momentum transfer (scattering force) for a single pulse time (Figure 2g and Figures S4–S10, Supporting Information). For lower scanning speeds (i.e., longer laser exposure time per unit distance), the photon momentum seems to be transferred directly or indirectly via precipitation‐redissolution of the nanoparticles/rods to the migrating monomers in the crystal lattice, causing them to move further (Figure 2h and Figures S6–S8, Supporting Information). This moving distance can even reach ≈1.5 µm at irradiation conditions of 265.8 mJ cm^−2^ and scan speed 0.01 mm s^−1^ at 5 MHz repetition rate (Figure S8, Supporting Information). For the precipitation‐redissolution process of NPs, amorphization channels resulting from strong plasmon‐lattice interactions can be observed near the nanorods in the crystal (Figure 2i). Although the heat accumulation between pulses due to the high repetition rate of the laser plays a crucial role in the system's evolution, the energy and momentum coupling from photons to the system throughout the processing is primarily mediated by plasmons. Given that the LSPR relaxation time (≈10 ns) is much shorter than the pulse interval (200 ns), the entire processing can still be conducted through multiple scans. This is scenario is consistent with multiple laser inscriptions at the same location (Figure S11, Supporting Information), meaning that depth control of the nanoparticles can be achieved by the energy (fluence) control at the irradiated area.

### 2.2 Hybrid Plasmonic Nanomaterials for Photonics Applications

**Figure 3:**
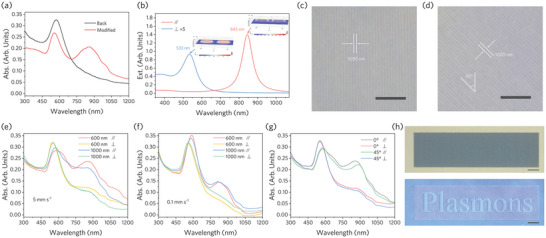
Tunable plasmon resonance modes and polarization dependence. The symbols “*∥*” and “⊥” represent that the incident light is parallel and perpendicular to the long axis of the synthesized Au nanorods, respectively. a) Measured absorption spectra of Au NPs in YAG, corresponding to the write parameter of 216.6 mJ cm^−2^ at a write speed of 5 mm s^−1^. b) Extinction cross section of Au nanorods calculated by finite element simulation. c,d) Top optical microscope view of the nanoparticle assembly arrays. Write energy of all arrays is controlled at 216.6 mJ cm^−2^. Scale bar: 10 µm. e,f) The absorption spectra of the arrays with periods of 600 and 1000 nm were written at scanning speeds of e) 5 mm s^−1^ and f) 0.1 mm s^−1^, respectively. g) Optical response of arrays with a period of 800 nm written in different directions at the scan speed of 5 mm s^−1^. h) Logos of “Plasmonic,” with laser‐write parameters of 264.8 mJ cm^−2^ 0.1 mm s^−1^ (character) and 216.6 mJ cm^−2^ 5 mm s^−1^ (background). Observed in reflection (top) and *∥*‐direction polarized transmission (bottom) modes. Scale bar: 10 µm.

Controllable NPs morphology and distribution define various plasmon resonance modes, which allows tailoring the optical response of the system. The measured absorption spectrum changes shown in **Figure** **3**a further reflect the distinct morphological characteristics of the NPs after laser processing. A new absorption peak of the modified NPs can be seen at a wavelength of about 850 nm, which comes from the plasmon resonance excited along the long axis of the nanorod (Figure S12a, Supporting Information). Compared with the resonance characteristics of isolated nanorod, in our experiments the plasmon coupling between adjacent nanorods result in a red shift of the near‐infrared resonance peak, which is further revealed by simulations (Figure 3b and Figure S12b–d, Supporting Information). Furthermore, some multipolar bonding modes can also be observed in the absorption spectra, originating from some NPs in close contact with each other (e.g., Figure 2e). Tuned plasmon resonances are demonstrated through wire‐by‐wire written nanoparticle assemblies as shown in Figure 3c,d, and the results are shown in Figure 3e–g.

In addition to the coupling of resonances between adjacent particles, plasmon hybridization between ordered arrays of assembled NPs can provide additional resonance modulation, such as the spectral broadening produced by polarized light excitation along the long axis of the nanorods in Figure 3e. This provides a way to independently modulate the internal and external coupling of plasmons in a nanostructure through laser parameters and patterning, thereby defining resonant modes. As shown in Figure 3g, further red‐shifting of the near‐infrared resonance peak is achieved by rotating the writing direction. It is known from the morphological characterization of the modified regions (e.g., Figure 2h) that slowing down the scanning speed reduces the aspect ratio of the nanorods, which can control the polarization dependence of the resonance (Figure 3f). Figure 3h demonstrates a simple example by achieving super‐resolution printing, in which the pattern can only obtain a clear image with high contrast when the illumination light is polarized parallel to the long axis of the nanorod.

**Figure 4:**
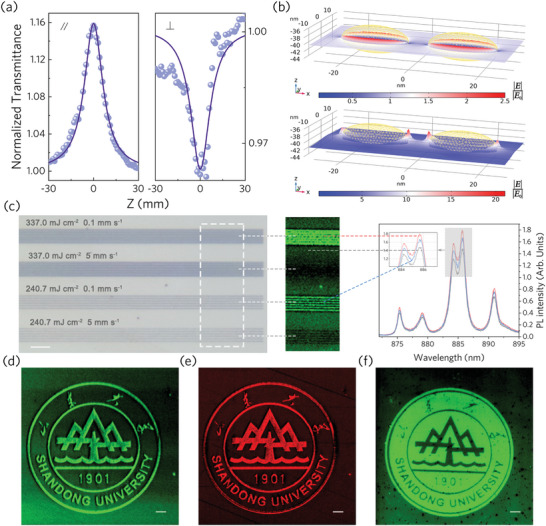
a) Open‐aperture Z‐scan results of YAG containing nanorods (1030 nm femtosecond laser excitation). The symbols “∥” and “⊥” represent that the incident light is parallel and perpendicular to the long axis of the synthesized Au nanorods, respectively. b) The near‐field distribution of plasmons excited along different axes of the nanorod at a wavelength of 1030 nm simulated by the finite element method. c) Top optical microscope view and PL measurements of laser inscribed nanoparticle assembly arrays with different parameters buried in Nd:YAG. PL mapping was acquired at 886 nm excited by light of 532 nm. Scale bar: 20 µm. d–f) The Shandong University logo consists of d,e) Au and f) Ag NPs in Nd:YAG displayed by PL at 886 nm excited by light of d,f) 532 nm and e) 633 nm wavelengths respectively. Scale bar: 20 µm.

Plasmon excitation along different axes of the nanorod allows encoding and transmission of polarization information, which may also produce optical nonlinearity modulated along different directions. The nonlinear optical properties of the obtained nanorod‐YAG hybrid material were characterized by the open‐aperture Z‐scan method. Linearly polarized light with a frequency of 1030 nm is used for detection. **Figure** **4**a shows the normalized transmittance of the modified region obtained by excitation with light polarized along the long axis with energy of 0.60 µJ and polarized along the short axis with energy of 0.83 µJ, respectively. Figure S13a, Supporting Information, supplements the results of polarization measurements along the long axis at energy 0.83 µJ, while no obvious signal was detected for both the unprocessed sample and pure YAG. Figure 4b indicates the plasmon near‐field excited at 1030 nm along different axes of the nanorod obtained by finite element simulation. For the case of light illumination along the long axis of the nanorod, the material exhibits typical saturated absorption (SA) characteristics, which originates from the ground state bleaching mechanism caused by the strong absorption of excited plasmons. The nonlinear absorption coefficient *β* is fitted to −510.5 cm GW^−1^ for 0.60 µJ pulse energy. Different from ordinary nanorods, the atomic‐level contact between NPs and YAG lattice will generate a potential barrier—a heterojunction—at the interface and affect the electronic state density,^[^ ^46^, ^47^ ^]^ thereby creating a new photon absorption mechanism. For the case of plasmons excited along the short axis (Figure 2b), the resonance peak corresponds to a higher photon energy (Figure S12a, Supporting Information) and there is not enough in‐band transition rate at 1030 nm to generate SA. However, the nanorods have a larger contact area with the lattice and a wider near‐field range along the short axis direction, which promotes phonon‐assisted multiphoton absorption. The coefficient *β* of this reverse‐saturable absorption (RSA) is fitted to 78.4 cm GW^−1^.

Because of the flexibility in a host matrix selection for ion implantation, this plasmonic nanolithography strategy can be well extended to practical applications in dielectric crystals with specific functions. For example, the PL measured after femtosecond laser direct writing of Nd‐doped YAG (Nd:YAG) containing Au NPs is shown in Figure 4c and Figure S13b–d (Supporting Information). It can be seen that the plasmon‐enhanced rare earth ion upconversion luminescence can be controlled by adjusting the LSPR mode of the NPs system. The enhanced PL originates from the stronger near field generated by the increase in particle size after irradiation. However, for the luminescence of Nd^3+^ ions, its emission spectrum overlaps with the near‐infrared absorption spectrum of nanorods (Figure 3a), which makes the fluorescence of the modified region of NPs with higher aspect ratio weakened instead. Nanoscale patterning‐controlled upconversion luminescence of rare earth ions allows applications in data storage, nano‐lasers, and compact optics. Figure 4d,e shows the luminescence at 886 nm of the logo of Shandong University composed of NPs in Nd:YAG under excitation at 532 and 633 nm wavelengths, respectively. We further evaluated the selection of elements constituting NPs, and the results showed that enhanced PL can also be achieved in Ag ion‐implanted Nd:YAG (Figure 4f and Figure S13e,f, Supporting Information).

**Figure 5:**
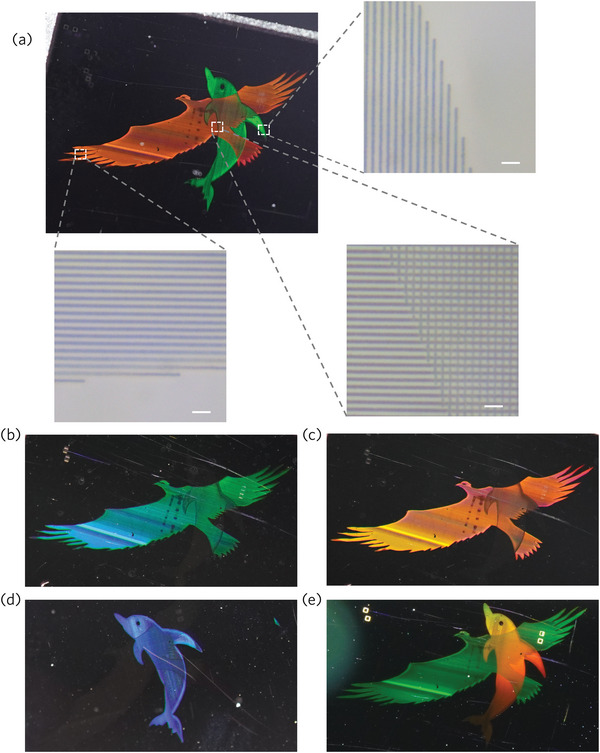
Photograph of structural color patterns formed by gratings of Au NPs in YAG. a) Structural color patterns of “Eagle” and “Dolphin,” and top optical view of their local nanostructures. Scale bar: 5 µm. b–d) Independent display of “Eagle” and “Dolphin” patterns. The viewing angle is fixed, and the colors of different patterns are adjusted separately by the incident angles of two independent beams of illumination light. e) Transmission structural color.

The interference and diffraction processes of small‐sized periodic structures exhibit a remarkable ability to generate vivid structural colors.^[^ ^48^, ^49^ ^]^ Through the precise manipulation of laser‐induced nanoparticle arrays that intersect seamlessly, intricate patterns can be inscribed in varying orientations, enabling the creation of structural colors with customizable plasmonic characteristics. These properties lend themselves to a diverse array of applications, including multidimensional information encoding, anti‐counterfeiting measures, polarization control, and modulated fluorescence. **Figure** **5** shows the structural colors of the “Eagle” and “Dolphin” patterns captured by the camera. The inset in Figure 5a shows the local structures that make up the pattern. By adjusting the angle and direction of the incident light, the angle‐dependent structural color and the independent display of “Eagle” and “Dolphin” are shown in Figure 5b–d (see Figure S14, Supporting Information, for more details). Additionally, the transparency of our sample under visible light allows for the transmission of structural colors, as depicted in Figure 5e. Notably, the plasmonic metamaterials embedded within solids, as opposed to surface‐synthesized structures or stripes, exhibit exceptional physicochemical stability and a strong affinity to the surrounding dielectric environment. This inherent stability and integration potential make them ideal candidates for incorporation into sophisticated opto‐electronic structures and devices, including fast optical switches of light‐controlled‐by‐light (Z‐scan) further advancing the field of photonics.

## 3 Conclusion and Outlook

In summary, our study showcases the precise incorporation of plasmonic NPs into the crystal lattice as hybrid plasmon materials, highlighting their potential applications in the field of optics. The ex‐situ reconstruction of NPs within the crystal lattice using femtosecond laser direct writing significantly expands the range of nanostructures achievable through laser processing. We elucidate the key mechanisms that govern the evolution of the system, with plasmon‐mediated photon energy deposition and momentum transfer via photon pressure playing crucial roles. This innovative process introduces a new concept of NPs’ formation harnessing scattering forces, which drive nanoclusters along the path of light propagation, facilitated by the crystalline orientation of the host lattice for smaller metallic clusters and by remelting and recrystallization. While the dynamics of this evolution are intricate and complex, the morphology and distribution of NPs can be effectively controlled through adjustments in single‐pulse energy, scanning speed, and energy deposition dose.

The organized assembly of nanoparticle systems in various states serves as the foundational elements, enabling the customized manipulation of optical responses and nanoscale information encoding, as demonstrated by multiple instances. Moreover, the revealed coupling mechanism identified in NP‐lattice hybrid structures paves the way for the development of plasmonic photonics applications rooted in the characteristics of dielectric crystals. By harnessing ion beams for the precise deposition of NPs with controlled depth in any substrate material, this approach harbors the capability to tailor on‐chip/in‐chip integrated micro‐nano photonic structures and devices with exceptional properties in functional materials/single crystal films. Since ion implantation provides sub‐surface (tens‐of‐nm) nano‐thin (tens‐of‐nm) regions of foreign metal ions and atoms, they can be further remorphed into different shapes and sizes using polarized fs‐laser direct write. This opens true nanoscale precision in the longitudinal direction of direct laser write, which is otherwise not achievable due to long axial extent even tightly focused laser pulses (depth of focus >> wavelength). Controlling crystal‐amorphous phases around nanoparticle by energy deposition using fs‐laser pulses allows harness photon pressure (photon wind) to position NPs within volumes with cross section exceeding the size of NPs by more than order of magnitude.

## 4 Experimental Section

### Sample Fabrication

Commercial YAG and Nd:YAG wafers with a size of 10 × 10 × 1.5 mm^3^ were optically polished for ion implantation. The Au/Ag ions were implanted into the YAG substrate by an ion‐implanter. The ion fluence for each sample is 3 × 10^16^ ions cm^−2^. The ion implantation surface and the crystal surface were at an angle of 7° to avoid channeling effects. The Au/Ag ions were randomly introduced into (Nd:)YAG and aggregated to synthesize NPs when the atomic concentration exceeded the solubility limit.

The samples were processed using a fiber chirped pulse amplification (FemtoYL‐10, YSL Photonics Ltd., China) system with a center wavelength of 1030 nm and a duration of 350 fs. The laser repetition rate was set to 5 MHz in the experiments. A half‐wave plate combined with a polarizing beam splitter was used to control the pulse energy. A 0.45 numerical aperture (NA) objective lens was used to focus the laser pulse below the sample surface near the surface, corresponding to a focal spot diameter of ≈2.8 µm (1.22 λ per NA). The working position of samples and the displacement velocity were manipulated by a computer‐controlled 6‐axis motorized stage (Hybrid Hexapod, Alio Industries Inc., USA) with a spatial resolution of 0.1 µm. All femtosecond laser processes were performed at standard atmospheric pressure and room temperature.

### Sample Characterization

Optical microscopic images of the processed area were captured by a homemade coaxial microscopy imaging system equipped on the femtosecond laser processing platform and a metallographic microscope (Carl Zeiss Microscopy GmbH, Germany). All micro‐nanomorphology observations in the modified area were performed by Talos F200X G2 (S)TEM (ThermoFisher Scientific Inc., USA), and the information resolution of TEM: 0.12 nm. The linear absorption in the wavelength range of 300–1200 nm was measured with a UV–VIS–NIR microspectrophotometer (20/30 PVTM, CRAIC Technologies Inc., USA) at room temperature.

Nonlinear characteristics were detected using a Gaussian beam with a beam waist radius of 50 µm, a pulse width of 400 fs, a repetition rate of 100 kHz, and a wavelength of 1030 nm. The normalized transmittance *T*_Norm_(*z*) of the sample measured in the open hole z‐scan experiment as a function of *z* can be described as:^[^ ^50^ ^]^

(1) $$T_{N\mathrm{orm}}\left(z\right)=1-\frac{\beta I_{0}L_{\mathrm{eff}}}{2\sqrt{2}\left(1+\frac{z^{2}}{z_{0}^{2}}\right)}$$

where *β* is the third‐order nonlinear coefficient, *I*_0_ is the peak intensity of the detection light, *L*_eff_ = (1‐e^−^ *^α^*_0_ *^L^*)/*α*_0_ is the effective thickness of the modified region, *α*_0_ is the linear absorption coefficient, *L* is the thickness of the modified region, *z*_0_ = π*ω* ^2^_0_/λ is the Rayleigh range, and *ω*_0_ is the beam waist radius.

All PL signals were collected by a confocal microprobe Raman spectrometer (Renishaw in Via, Renishaw PLC., UK) at room temperature. The excitation wavelengths used were 532 and 633 nm. The structural color of the sample under visible light illumination was captured by a single‐lens reflex camera (ILCE‐6400, SONY Corp., Japan) with the ISO‐800.

### Optical Simulations

The optical forces acting on the particles are calculated from the Maxwell stress tensor, which is described as:^[^ ^51^, ^52^ ^]^

(2) $$\bar{\bar{T}}=\frac{1}{2}Re\left[\varepsilon EE^{*}+\mu HH^{*}-\frac{1}{2}\left(\varepsilon EE^{*}+\mu HH^{*}\right)\bar{\bar{I}}\right]$$

where $\bar{\bar{I}}$ is the unit tensor, **E** and **H** are the complex electric and magnetic fields, and *ε* and *µ* are the dielectric constant and magnetic permeability of the matrix.

Numerical simulation of the optical properties of NPs in YAG matrix was performed using the commercial software COMSOL Multiphysics. Based on the actual nanoparticle distribution and morphology, scatterer models composed of five NPs and nanorods were established respectively. For the five NPs model, the incident light is incident along the z‐axis and polarized along the y‐axis. The optical force is obtained by integrating the tensor in Equation 2 over a closed surface around the particle. In all simulations, the refractive index of Au NPs was defined by the Lorentz‐Drude model. Electromagnetic waves were incident vertically (along the z‐axis) from the air domain onto the YAG containing NPs. Apply perfectly matched layers around the physical area to simulate a domain with open boundaries.

## Conflict of Interest

The authors declare no conflict of interest.

## Supporting information

Supporting Information

## Data Availability

The data that support the findings of this study are available from the corresponding author upon reasonable request.
