# Supplementary material for: Ultrafast Laser Manipulation of In‐Lattice Plasmonic Nanoparticles
Source: Adv Sci (Weinh). 2024 Jul 18;11(38):2402840. doi: 10.1002/advs.202402840 (PMC11481187; doi:10.1002/advs.202402840)
Supplement: Supplementary file 1 — Supporting Information [file ADVS-11-2402840-s001.docx]

Supporting Information

**Ultrafast laser manipulation of in-lattice plasmonic nanoparticles**

*Han Zhu, Lingrui Chu, Hengyue Lv, Qingchuan Ye, Saulius Juodkazis*, and Feng Chen**

**This file includes:**

Figs. S1 to S14

Tables S1

The precursor YAG embedded with Au/Ag NPs and atomic impurities for subsequent ultrafast laser processing was prepared by ion implantation, and the detailed preparation parameters are shown in **Table S1**. In this work, except for PL and structural color, all processing, characterization and testing were completed on sample 1. The patterned structural colors were drawn on sample 4 to verify that our method is robust to the control of implantation energy.

Similar laser irradiation experiments were performed on the back side of sample 1 (pure YAG without Au NPs) as shown in **Figure S1**. It can be seen that the average pulse energy used for plasmonic nanolithography is well below the threshold of pure YAG modification. **Figure S2** shows the morphology of the single crystal region and implanted layer in YAG after ion implantation of sample 1. The thickness of the implanted layer and the distribution of NPs therein depend on the range and distribution of ions in YAG, which can be simulated by the Stop and Range of Ions in Matter (SRIM) code as shown in **Figure S3**a-c. Figure S3d shows that the lateral gradient force between nanoparticles increases as the spacing decreases.

**Figure S4-S10** systematically show the morphology and structure of the modified area after processing with different laser parameters. It can be clearly seen that compared with Figure 2b (Figure S4b, S6b, S7c, S9c), the size of the NPs in the implanted layer after laser irradiation increases. **Figure S11** shows the morphological characterization of multiple laser inscriptions at the same location. The pulse energy was fixed at 264.8 mJ cm^-2^, and the first modification layer was formed by writing line by line without intervals at a scanning speed of 5 mm s^-1^, and then the second modification was performed at a speed of 0.1 mm s^-1^.

**Figure S12**a supplements the measured polarization-dependent absorption spectra of Au nanorods in YAG. The plasmon resonance characteristics of single nanorods in YAG simulated by finite element are shown in Figure S12b-d. **Figure S13** and **S14** supplement the results on nonlinearity, PL, and structural color achieved by plasmonic nanoparticle arrays.

Table S1. Parameters of sample preparation.

| No. | Implanted ions | Fluence [ions cm^−2^] | Energy [keV] | Crystal |
| --- | --- | --- | --- | --- |
| 1 | Au^+^ | 3×10^16^ | 125 | YAG |
| 2 | Au^+^ | 3×10^16^ | 125 | Nd:YAG |
| 3 | Ag^+^ | 3×10^16^ | 200 | YAG |
| 4 | Au^+^ | 3×10^16^ | 100 | YAG |


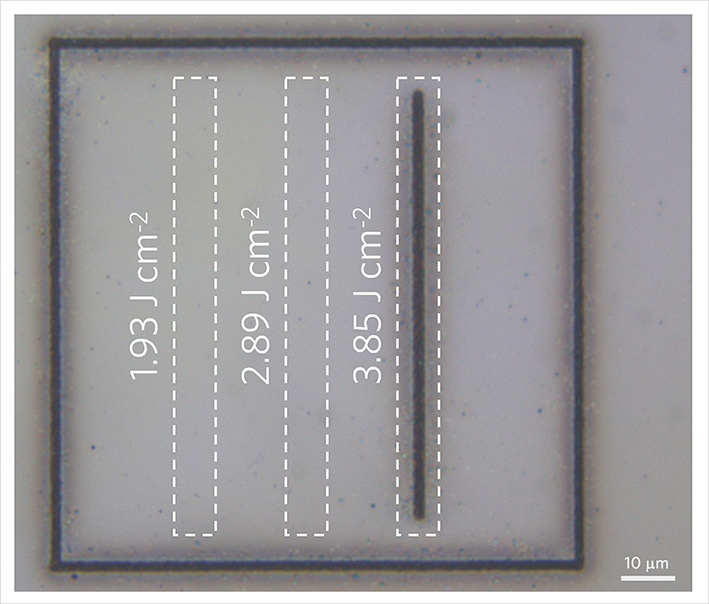


Figure S1. Femtosecond laser direct writing of pure YAG at different pulse energies. The laser repetition frequency is 5MHz and the writing speed is 5 mm s^-1^.


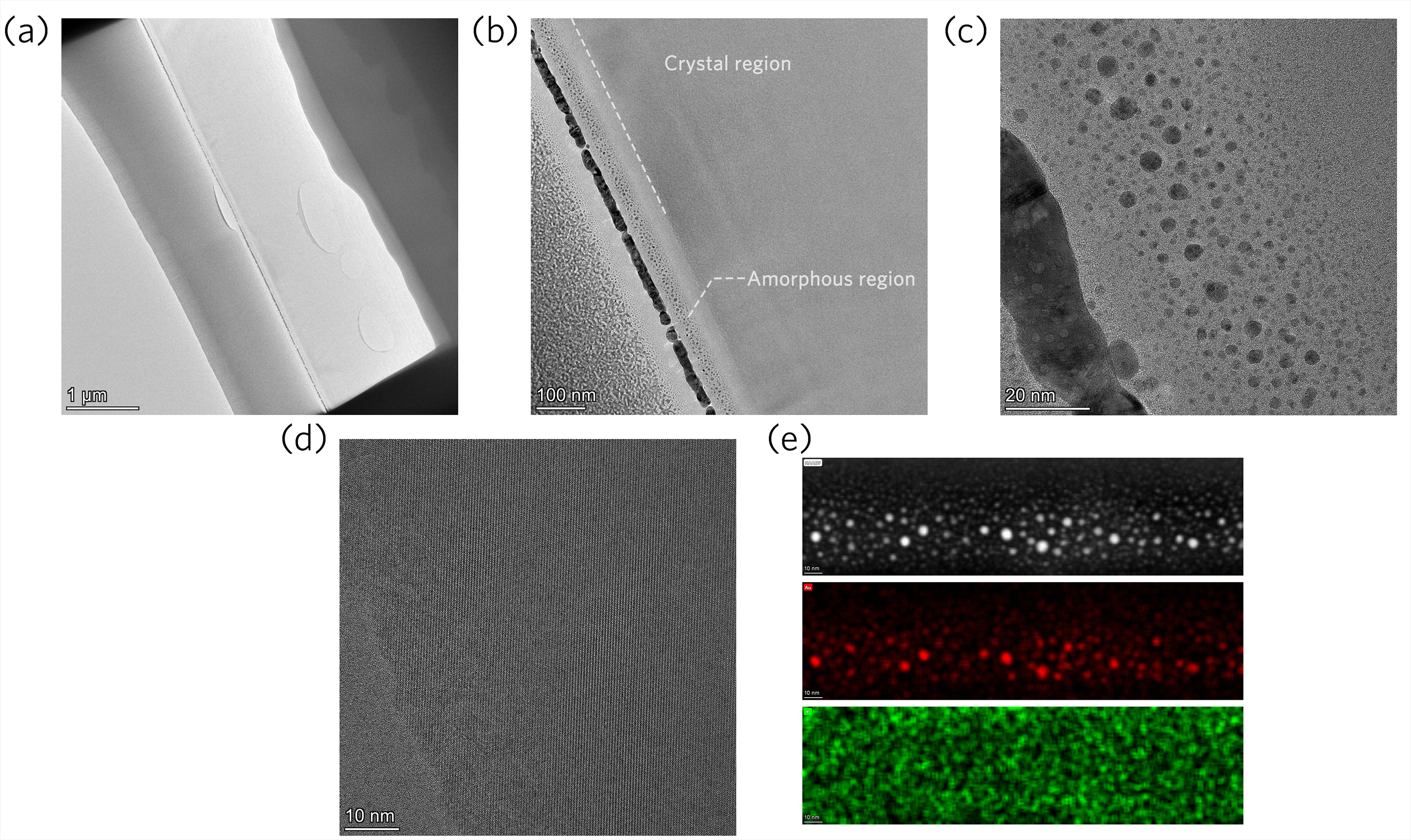


Figure S2. Morphological characterization of YAG implanted layer after ion implantation (before laser irradiation). a-d) Cross-sectional TEM image (a) and HRTEM images (b-d) of the YAG implanted layer, corresponding to Figure 1b-d. e) HAADF image and element mapping of Au NPs in YAG.


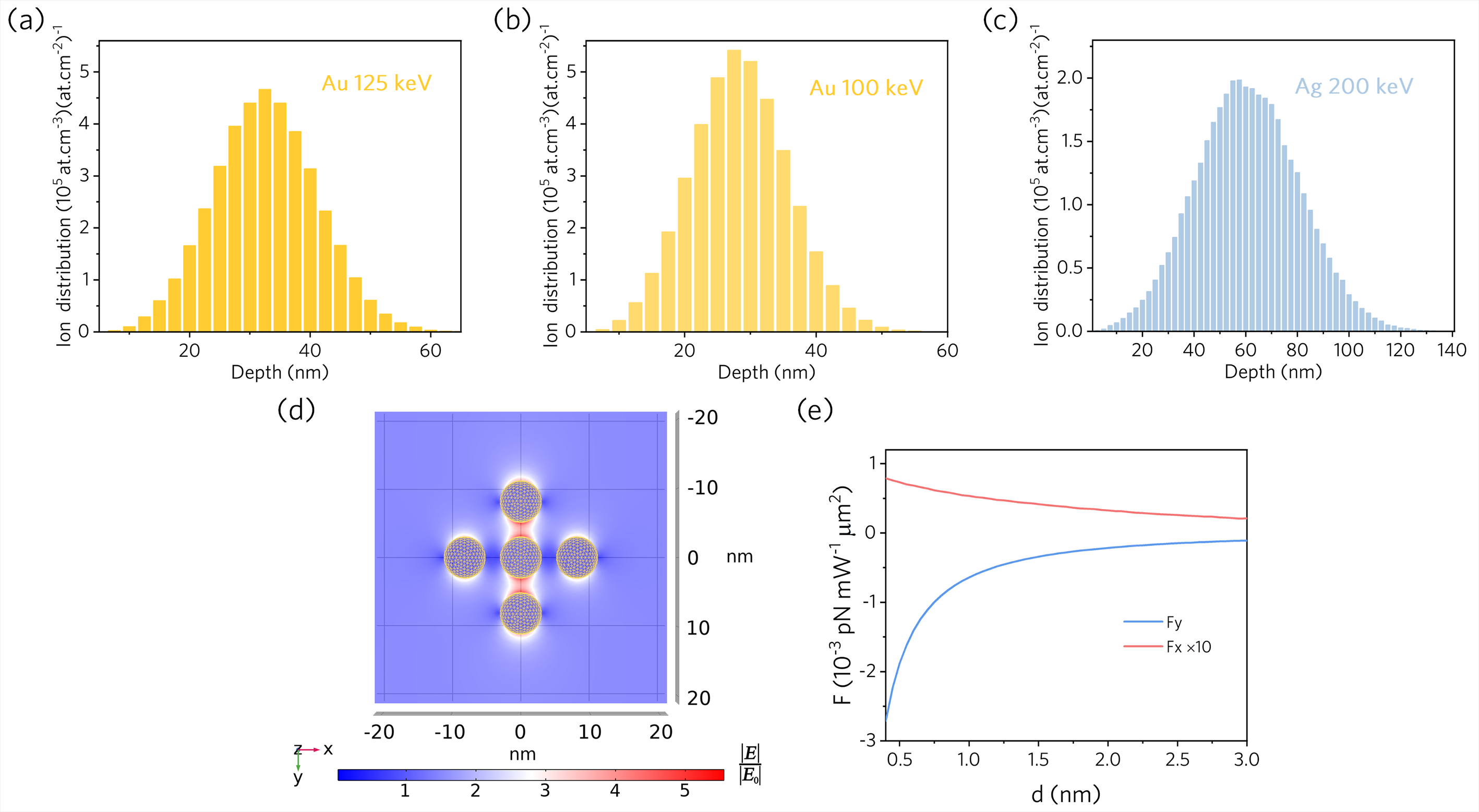


Figure S3. a-c) Ion distribution of different implant energies and different elements in YAG calculated by SRIM. (d) Numerical calculation of the near field of NPs in YAG, corresponding to Figure 1g. (e) Optical force of NPs in YAG as a function of particle distance *d*, corresponding to Figure 1f-h.


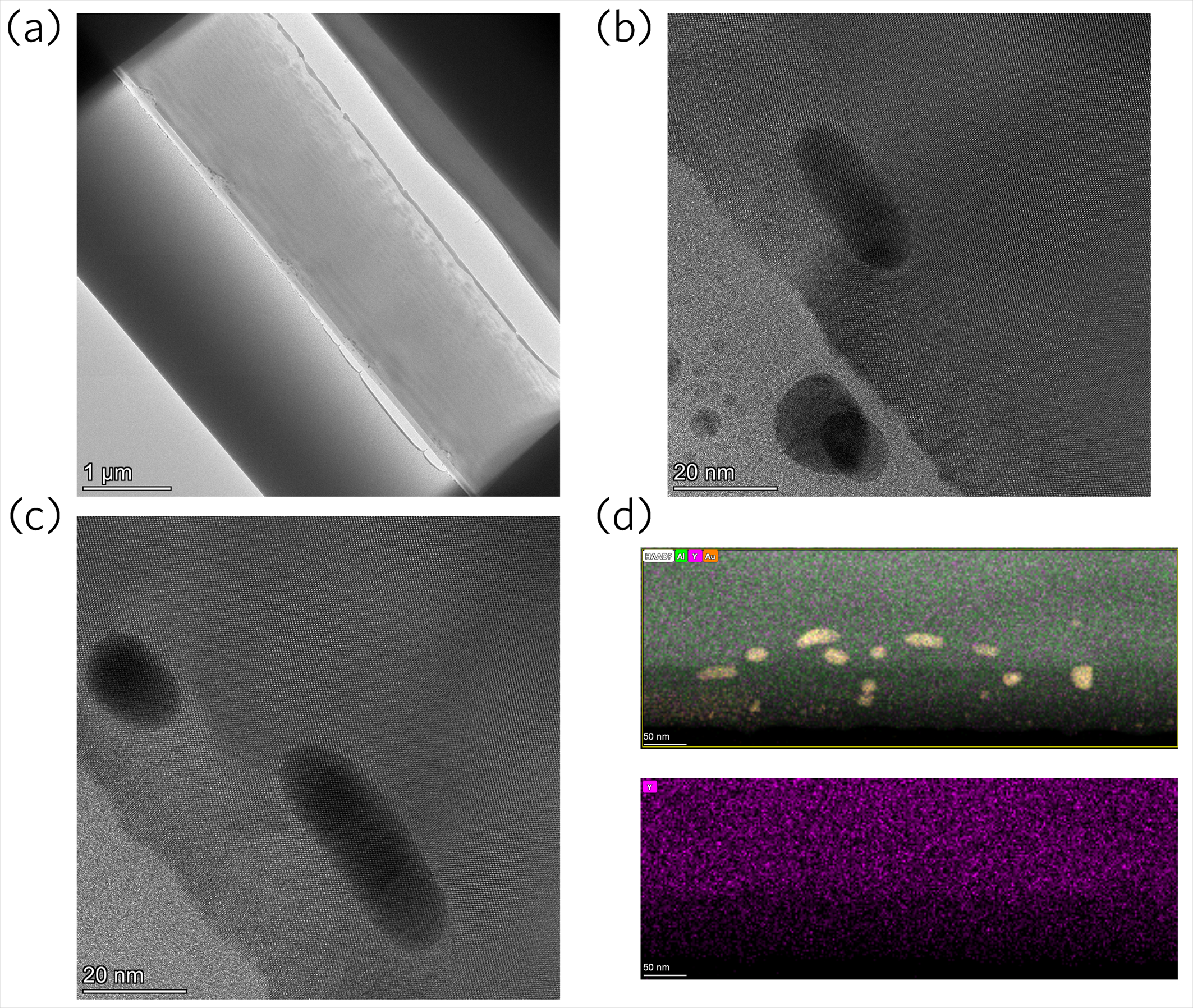


Figure S4. Morphological characterization of YAG containing Au NPs after laser irradiation of 216.6 mJ cm^-2^ 5 mm s^-1^. a-c) Cross-sectional TEM (a) and HRTEM (b,c) images of implanted layer and single crystal region. d) HAADF image and element mapping of the laser irradiated area.


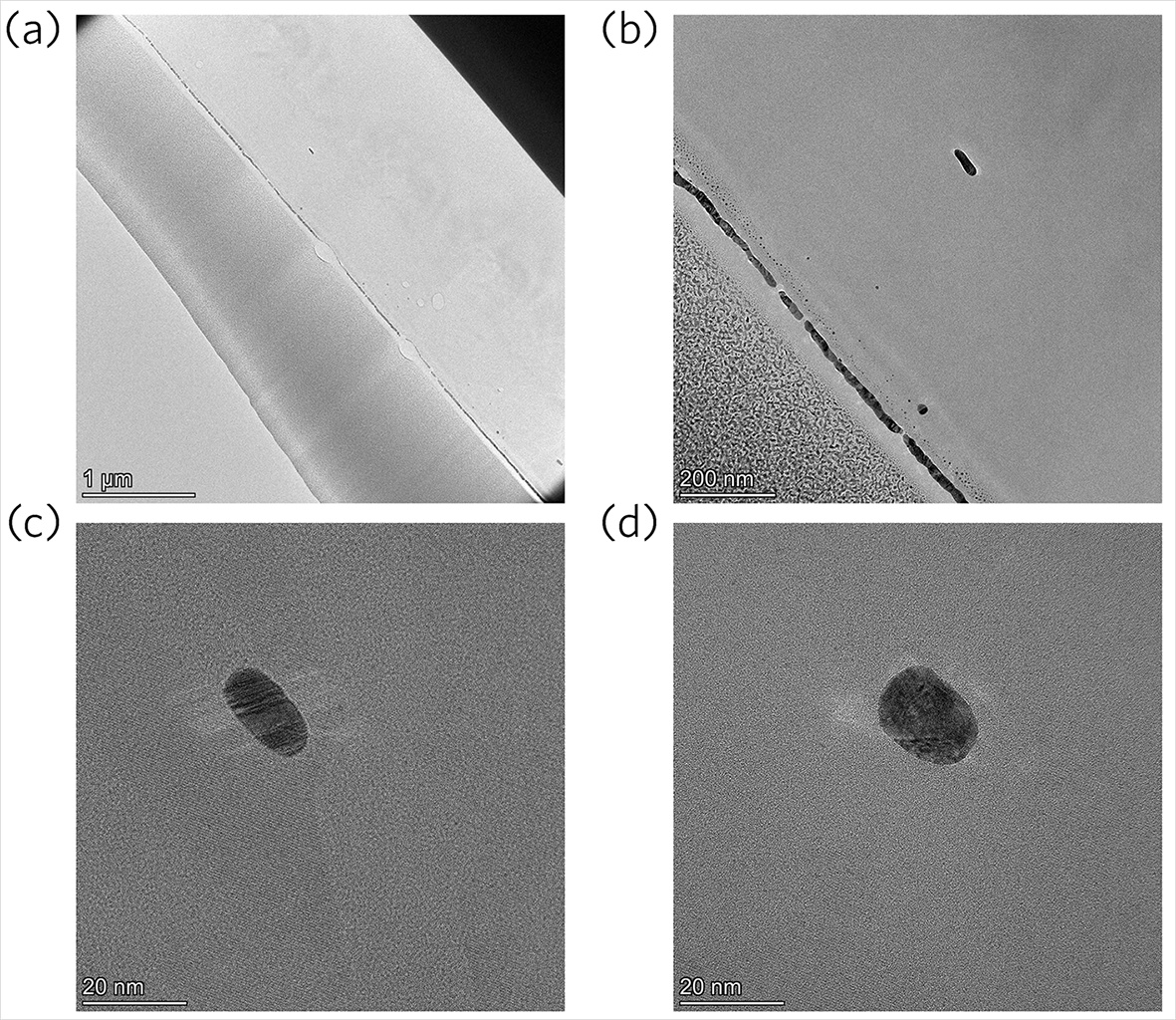


Figure S5. Morphological characterization of YAG containing Au NPs after laser irradiation of 216.6 mJ cm^-2^ 0.1 mm s^-1^. a,b) Cross-sectional TEM images. c,d) Cross-sectional HRTEM images of Au NPs. Amorphization of the prepared area is due to too thin FIB sectioning.


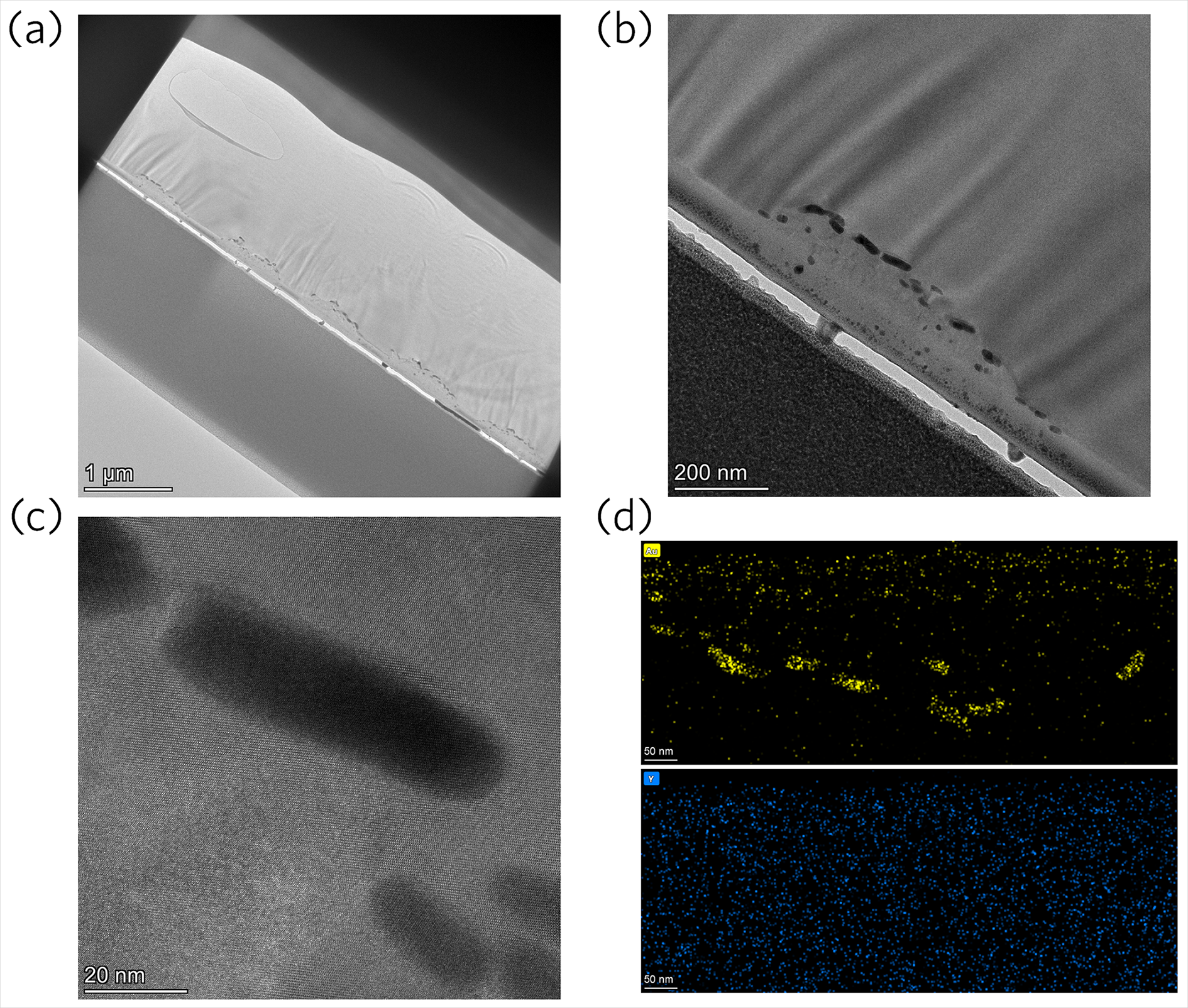


Figure S6. Morphological characterization of YAG containing Au NPs after laser irradiation of 264.8 mJ cm^-2^ 5 mm s^-1^. a,b) Cross-sectional TEM images of implanted layer and single crystal region. c) Cross-sectional HRTEM image of single crystal region. d) Element mapping of the laser irradiated area.


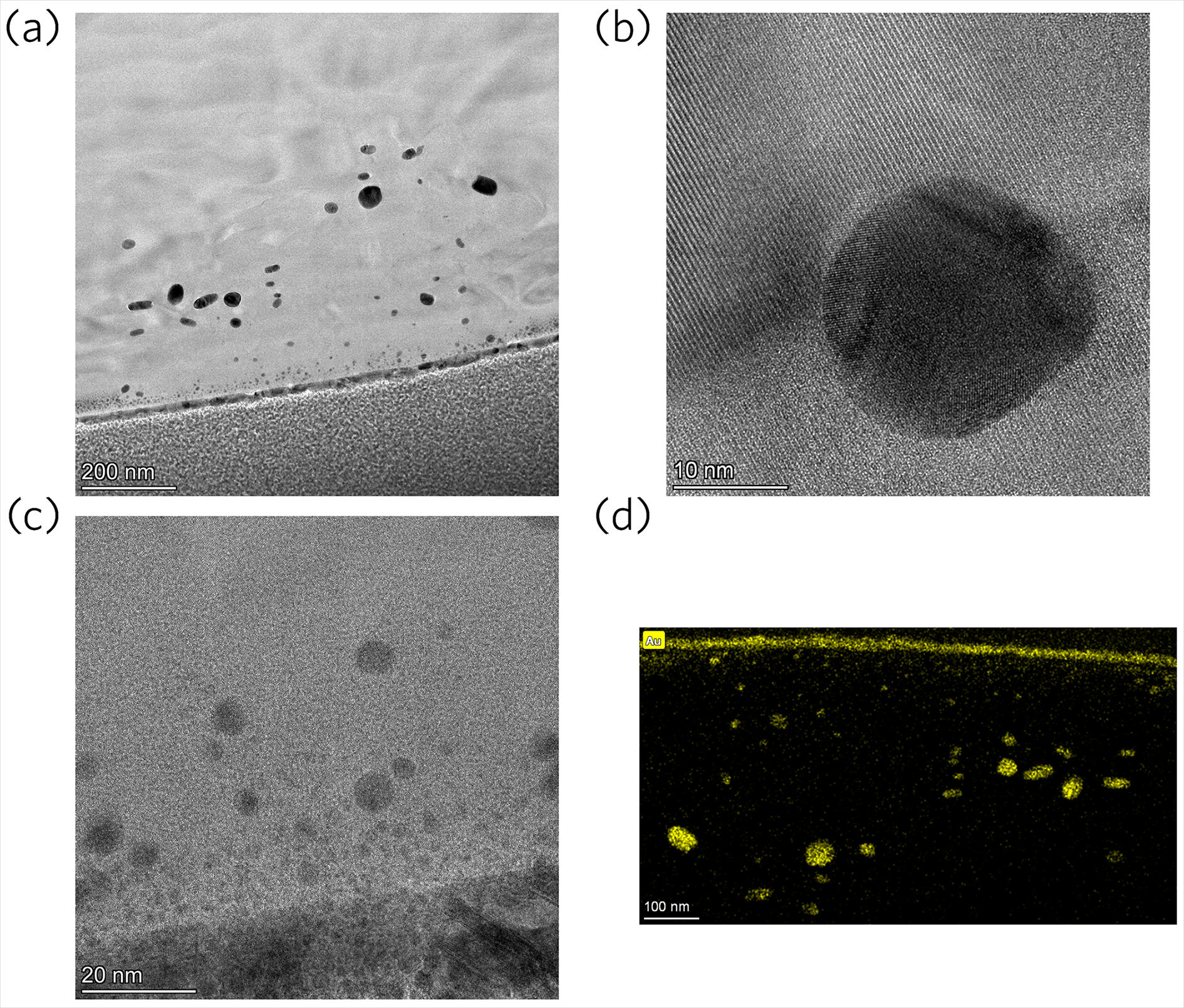


Figure S7. Morphological characterization of YAG containing Au NPs after laser irradiation of 264.8 mJ cm^-2^ 0.1 mm s^-1^. a-c) Cross-sectional TEM (a) and HRTEM (b,c) images of implanted layer (c) and single crystal region (b). d) Element mapping of the laser irradiated area.


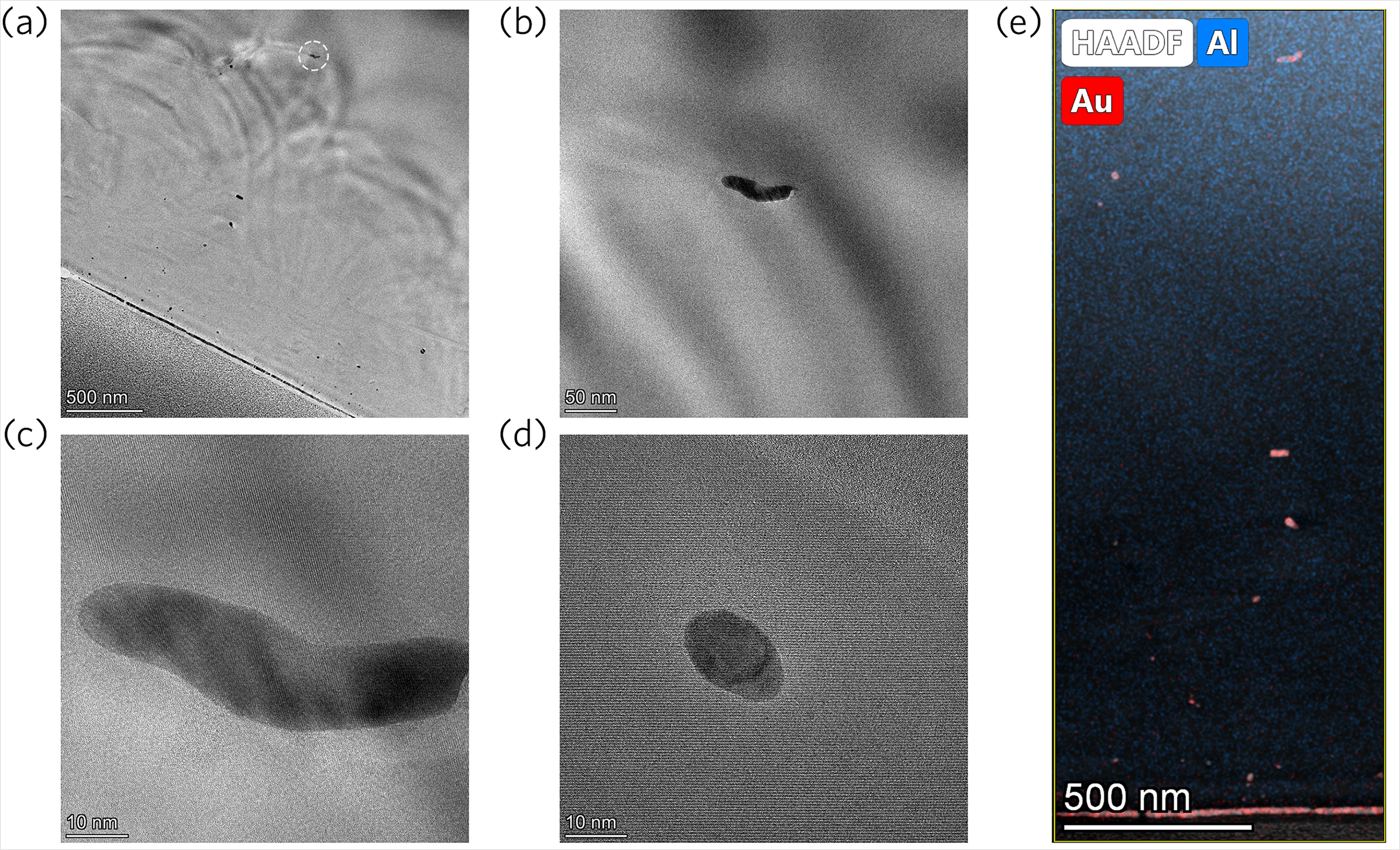


Figure S8. Morphological characterization of YAG containing Au NPs after laser irradiation of 264.8 mJ cm^-2^ 0.01 mm s^-1^. a) Cross-sectional TEM images of implanted layer and single crystal region. b) Cross-sectional TEM image of the selected area in (a). c,d) Cross-sectional HRTEM image of single crystal region containing NPs. Partial amorphization in the prepared area is due to too thin focused ion beam (FIB) sectioning. e) HAADF image and element mapping of the laser irradiated area.


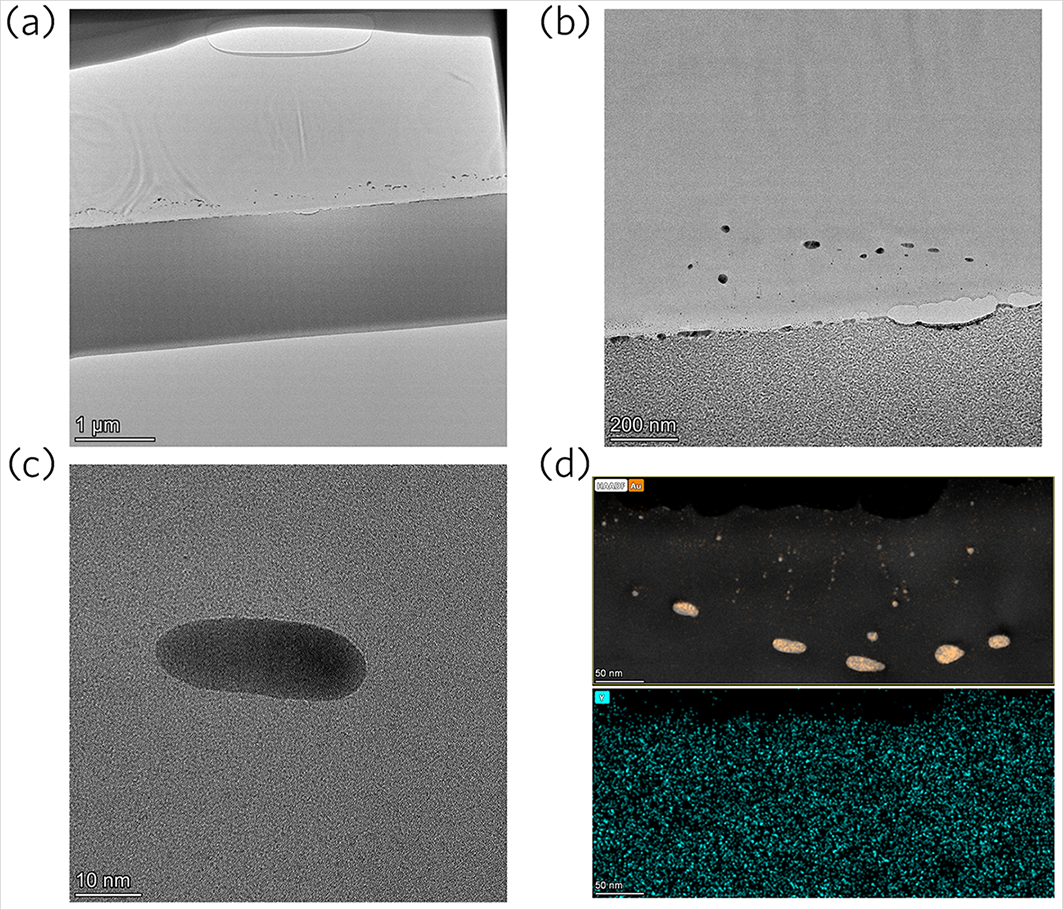


Figure S9. Morphological characterization of YAG containing Au NPs after laser irradiation of 337.0 mJ cm^-2^ 5 mm s^-1^. a,b) Cross-sectional TEM images. c) Cross-sectional HRTEM images of Au NPs. Amorphization of the prepared area is due to too thin FIB sectioning. d) HAADF image and element mapping of the laser irradiated area.


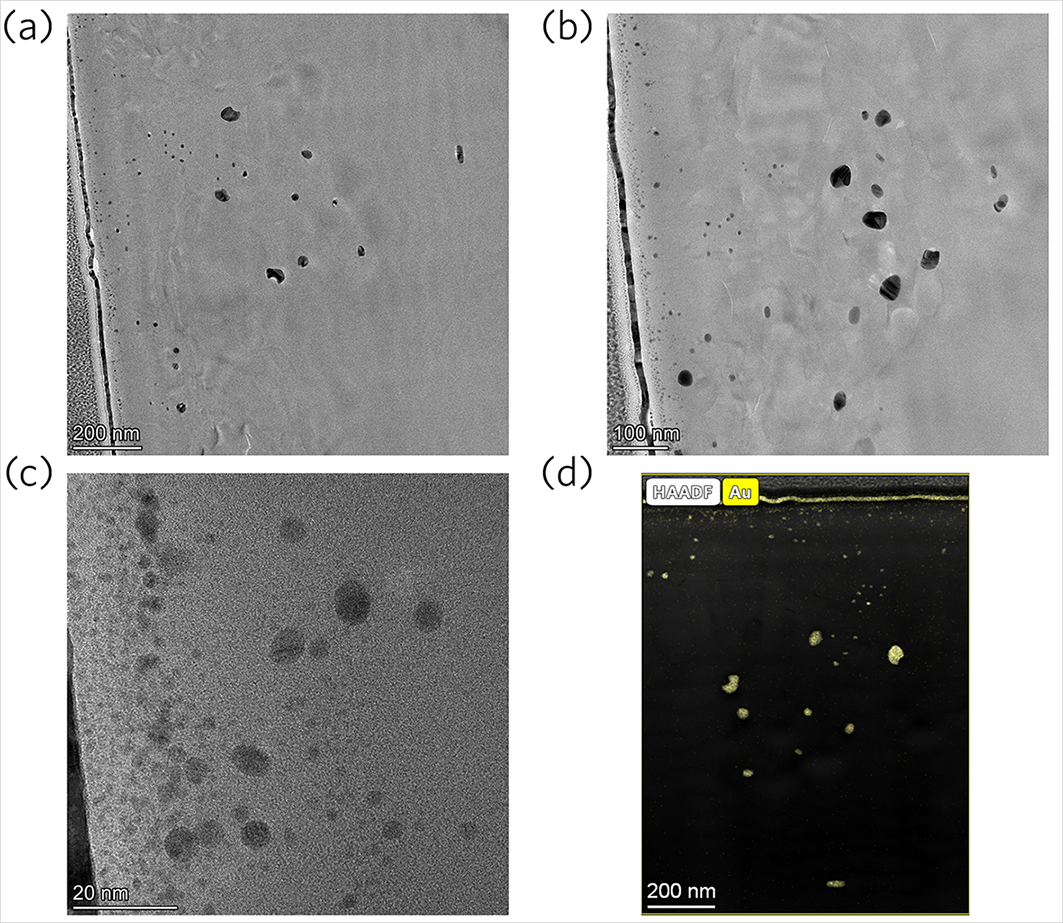


Figure S10. Morphological characterization of YAG containing Au NPs after laser irradiation of 337.0 mJ cm^-2^ 0.1 mm s^-1^. a,b) Cross-sectional TEM images. c) Cross-sectional HRTEM images of implanted layer. d) HAADF image and element mapping of the laser irradiated area.


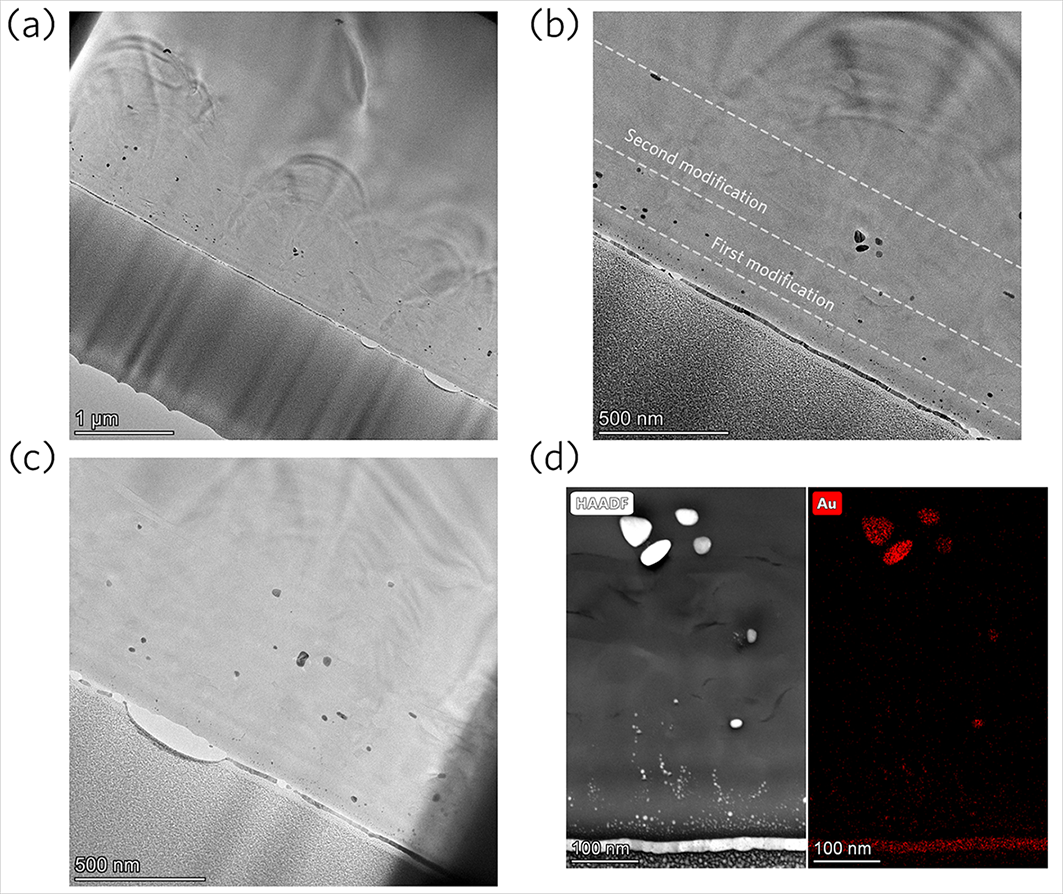


Figure S11. Morphological characteristics of YAG containing Au NPs after multiple laser inscriptions at the same location. The pulse energy is 264.8 mJ cm^-2^, and the scanning speed is 5 mm s^-1^ and 0.1 mm s^-1^ in sequence. a-c) Cross-sectional TEM images. d) HAADF image and element mapping of the laser irradiated area.


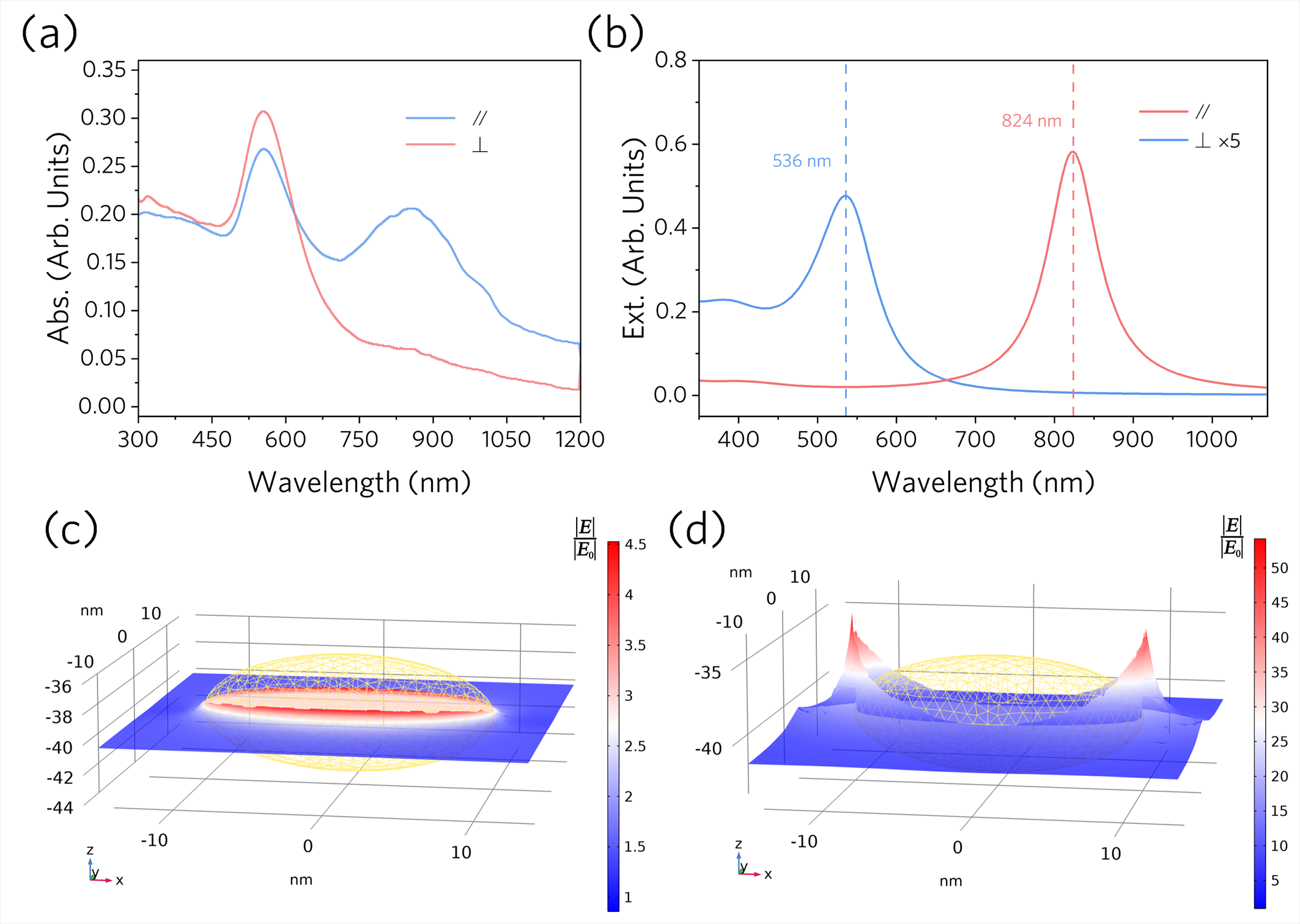


Figure S12. a) Measured absorption spectrum of Au NPs in YAG after laser irradiation, corresponding to the case in Figure 3a. b) Extinction cross section of a single Au nanorod calculated by finite element simulation. c,d) The near-field distribution of plasmons excited along different axes of the nanorod at wavelengths of 536 nm (c) and 824 nm (d) simulated by the finite element method.


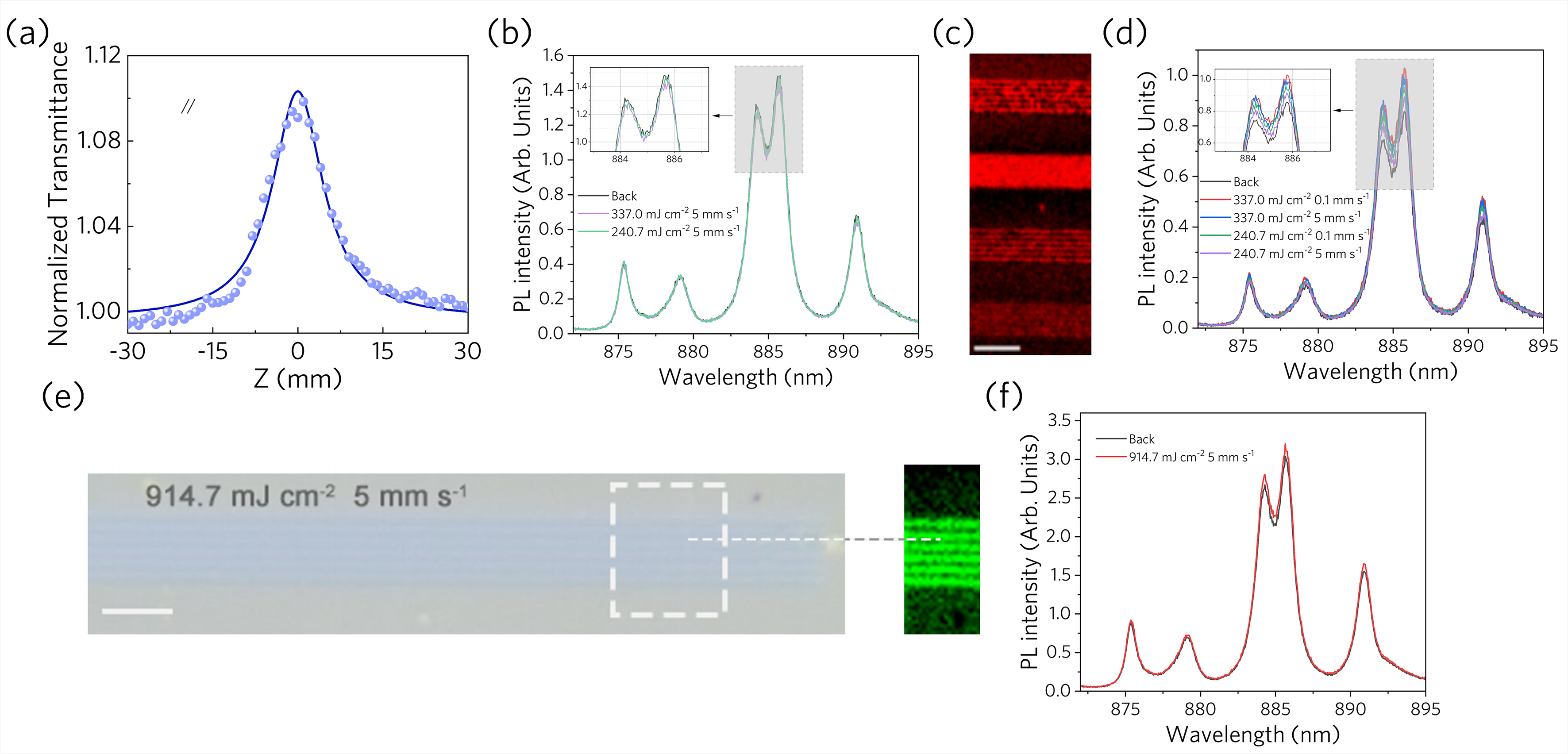


Figure S13. a) Open-aperture Z-scan results of YAG containing nanorods (1030 nm femtosecond laser excitation with energy of 0.83 μJ). b-d) PL measurements corresponding to the laser inscribed nanoparticle assembly arrays of Figure 4c. The excitation lights are 532 nm (b) and 633 nm (c,d) respectively. PL mapping was acquired at 886 nm excited by light of 633 nm. Scale bar: 20 μm. e,f) Top optical microscopy view and PL measurements of laser inscribed nanoparticle assembly arrays buried in Ag ion-implanted Nd:YAG. PL mapping was acquired at 886 nm excited by light of 532 nm. Scale bar: 20 μm.


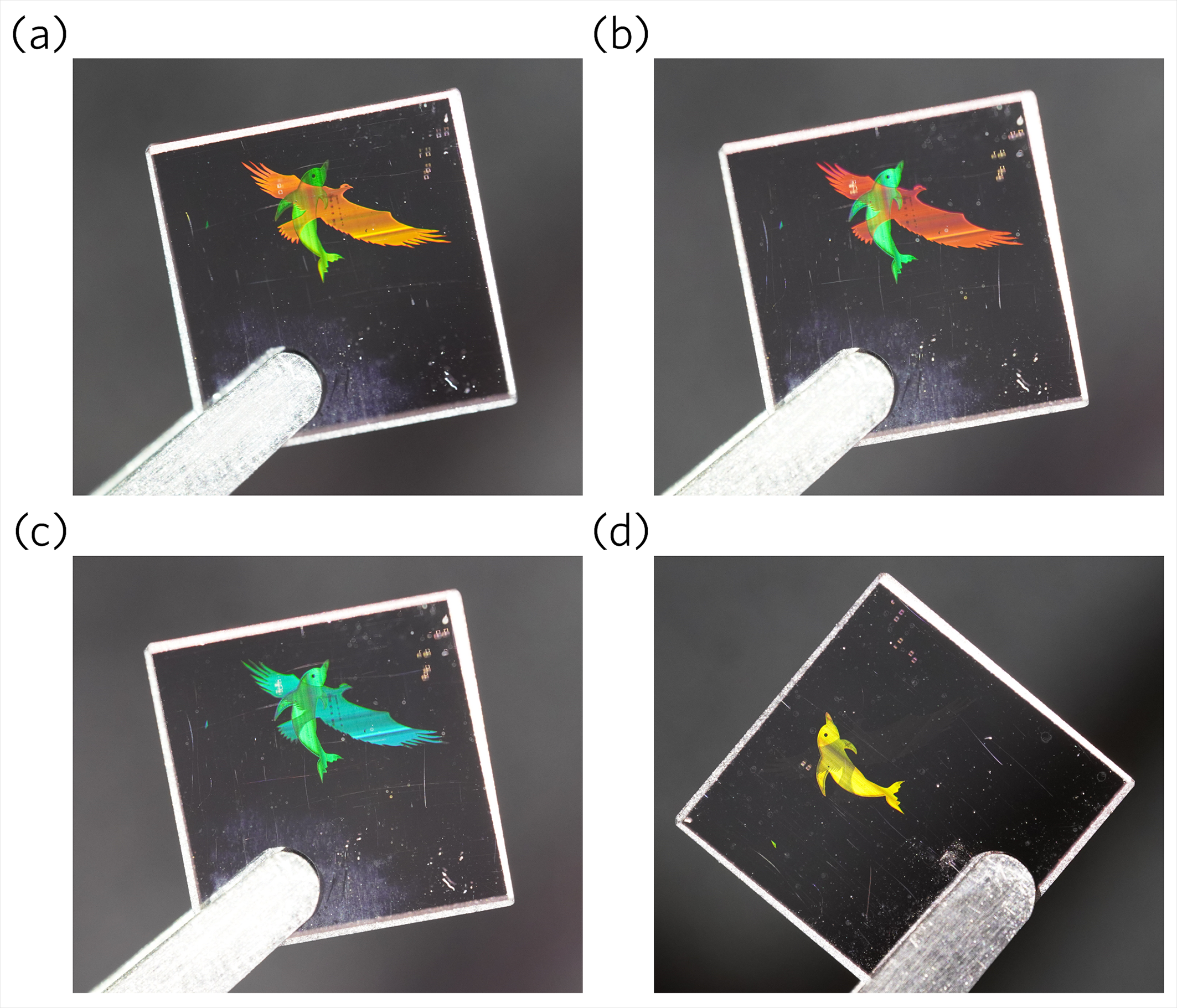


Figure S14. Camera captures structural color patterns formed by Au NPs gratings in YAG. a-c) Structural color patterns of "Eagle" and "Dolphin", the colors of which can be modulated independently of each other (b,c). d) Independent display of "dolphin" pattern.
